# Assessment of HIV-1 entry inhibitors by MLV/HIV-1 pseudotyped vectors

**DOI:** 10.1186/1742-6405-2-7

**Published:** 2005-09-12

**Authors:** Sandra Siegert, Sonja Thaler, Ralf Wagner, Barbara S Schnierle

**Affiliations:** 1 Georg-Speyer-Haus, Institute for Biomedical Research, Paul-Ehrlich-Strasse 42-44, D-60596 Frankfurt am Main, Germany; 2 Paul-Ehrlich Institute, Abt. 2/01, Paul-Ehrlich Strasse 51-59, D-63225 Langen, Germany; 3 Department of Medicine III, Johannes Gutenberg University, 55101 Mainz, Germany; 4 Institute of Medical Microbiology and Hygiene, University of Regensburg, Franz-Josef-Strauss Allee 11, 93053 Regensburg, Germany; 5 Friedrich Miescher Institute, Maulbeerstrasse 66, CH-4058 Basel, Switzerland

## Abstract

**Background:**

Murine leukemia virus (MLV) vector particles can be pseudotyped with a truncated variant of the human immunodeficiency virus type 1 (HIV-1) envelope protein (Env) and selectively target gene transfer to human cells expressing both CD4 and an appropriate co-receptor. Vector transduction mimics the HIV-1 entry process and is therefore a safe tool to study HIV-1 entry.

**Results:**

Using FLY cells, which express the MLV gag and pol genes, we generated stable producer cell lines that express the HIV-1 envelope gene and a retroviral vector genome encoding the green fluorescent protein (GFP). The BH10 or 89.6 P HIV-1 Env was expressed from a bicistronic vector which allowed the rapid selection of stable cell lines. A codon-usage-optimized synthetic *env *gene permitted high, Rev-independent Env expression. Vectors generated by these producer cells displayed different sensitivity to entry inhibitors.

**Conclusion:**

These data illustrate that MLV/HIV-1 vectors are a valuable screening system for entry inhibitors or neutralizing antisera generated by vaccines.

## Background

The acquired immunodeficiency syndrome (AIDS) was first described about 20 years ago. Since then almost 20 million people have died from human immunodeficiency virus (HIV-1) infection and 42 million are infected with. New drugs and an effective vaccine are urgently needed. In particular, new drugs that block the HIV type 1 (HIV-1) entry into host cell have clear advantages over the currently used drugs. They should abrogate the establishment of a productive infection and consequently could diminish the chances of HIV-1 developing resistance. Furthermore, a vaccine that prevents AIDS should elicit broadly cross-reactive neutralizing antibodies to prevent infection. A safe and simple assay for measuring neutralizing activities against different HIV-1 strains is critical for the development of such a vaccine or entry inhibiting drugs.

We previously generated a retroviral vector which specifically transfers genes into human CD4+ cells [1,2]. This vector was derived by pseudotyping murine leukemia virus (MLV) capsid particles with a variant of the HIV-1 envelope protein (Env) containing the surface glycoprotein gp120-SU and a carboxyl-terminally truncated transmembrane (TM) protein with only 7 cytoplasmic amino acids. HIV-1 Env facilitates vector attachment to target cells and membrane fusion, which is initiated by the interaction of HIV-1 Env with the CD4 receptor molecule on the surface of the target cell. CD4 binding induces a conformational change in the envelope glycoprotein and allows the binding of a co-receptor of the chemokine receptor family [3]. The co-receptor usage is virus strain dependent: R5 viruses, which infect monocytes and macrophages, use CCR5 and X4 viruses, which infect T cell lines, use CXCR4. X4R5 strains can use CXCR4 as well as CCR5 for entry. The transfer of a marker gene by MLV/HIV-1 vectors is therefore a safe and simple method to assay entry mediated by HIV-1 Env and can be used to evaluate HIV-1 entry inhibitors, such as small molecules or neutralizing antibodies in sera of vaccinated animals or patients.

Here, we optimized production of the MLV/HIV-1 vector to allow analysis of different HIV-1 Envs and demonstrate that the responsiveness to viral entry inhibitors was dependent on the HIV-1 strain the Env was derived from. This illustrates that MLV/HIV-1 pseudotyped vectors are useful tools for analyzing HIV-1 entry.

## Results and discussion

### Generation of a stable producer cell line encoding the 89.6 P HIV-1 Env

**Figure 1:**
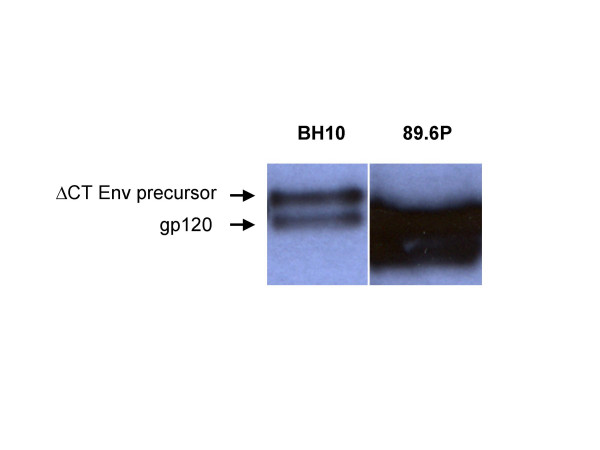
Expression of the 89.6 P HIV-1 Env. 293T cells were transfected with 3 μg plasmid DNA and cell lysates were analyzed after two days for HIV-1 Env expression by Western blot analysis. The two forms of Env are indicated as gp140 (C-terminally truncated precursor) and gp120 (SU).

We and others have previously reported that MLV capsids can be pseudotyped with cytoplasmatically truncated variants of the HIV-1 or HIV-2 envelope glycoproteins possessing only 7 cytoplasmic amino acids. These MLV/HIV pseudotyped vectors have the HIV host range [4,1,5]. We used a X4 HIV-1 Env variant (BH10) for pseudotyping, which restricted vector entry to CD4 and CXCR4 receptor-positive cells [2]. HIV-1 Env was expressed from an expression construct that also encoded Rev, which is required to transport the rev responsive element (RRE)-containing *env *mRNA from the nucleus to the cytoplasm. In the present study we evaluated a codon-usage-optimized HIV-1 *env *gene that encodes the truncated Env variant of the X4R5 89.6 P HIV-1 isolate and lacks *rev *sequences. Western blot analysis of transfected 293T cells showed that the change from lentiviral to mammalian codon usage allowed high HIV-1 Env protein expression in the absence of Rev (Figure 1). The 89.6 P Env showed different migration in polyacrylamide gels from the BH10 isolate, which might be caused by different glycosylation patterns of the protein and reflects strain-specific differences in Env.

We previously constructed a MLV/HIV-1 producer cell line based on FLY cells [6], which expresses the HIV-1 Env of the X4 HIV-1 BH10 strain and a retroviral vector encoding the green fluorescent protein (GFP) [2]. These cells are further referred to as FLY-HIV-87-GFP cells. HIV-1 Env protein expression is driven by the strong human elongation factor 1α promoter and stable clones were selected via the puromycin resistance gene (*pac*) encoded on the bicistronic messenger RNA.

We cloned a codon-usage-optimized 89.6 P HIV-1 Env into this vector and stable cell clones were rapidly isolated by puromycin selection. Protein expression of HIV-1 Env was ensured by expansion of the cells in the continued presence of puromycin. A single clone was further transduced with a GFP-encoding retroviral vector to obtain the producer cell line FLY-syn-GFP.

### Characterization of vectors particles derived from FLY-HIV-87-GFP or FLY-syn-GFP

**Figure 2:**
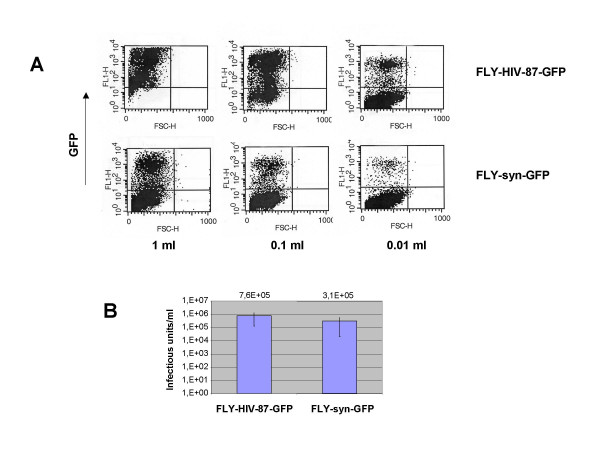
Titer of MLV/HIV-1 pseudotyped vector particles released from producer cells. A: NIH3T3-CD4/X4 cells (1 × 10^5^) were incubated with 1, 0.1 or 0.01 ml supernatant from producer cells in a total volume of 1 ml. For titer determination, the number of GFP+ cells (upper left) was determined. B: Titers were calculated by measuring the percentage of GFP-positive cells after transduction. Values are the average of 5 experiments.

**Figure 3:**
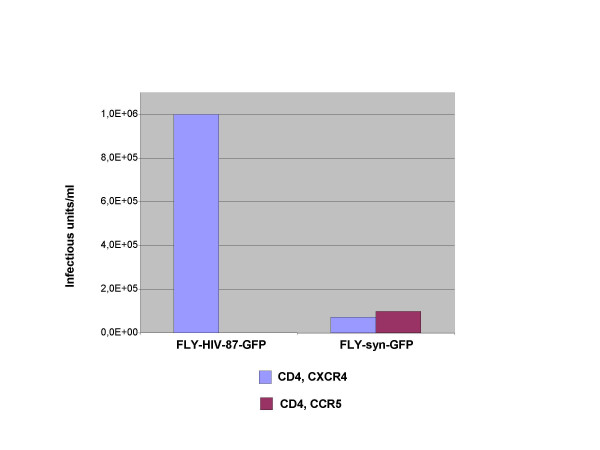
Co-receptor usage of MLV/HIV-1 vectors. NIH3T3 cells expressing the CD4 receptor and either CXCR4 or CCR5 were transduced with vector particles derived from FLY cell lines, and the titers were calculated by measuring the percentage of GFP-positive cells. The X4R5 89.6 P Env in vector particles derived from FLY-syn-GFP cells allowed the transduction of both target cell lines.

The characterization of the two producer cell lines was started by determining the amount of infectious retroviral vector particles released from the producer cells. Infectious particles can be easily detected by the transfer of the *gfp *gene. NIH3T3 CD4/CXCR4 cells that express the CD4 and CXCR4 receptors were transduced with supernatants derived from FLY-HIV-87-GFP or FLY-syn-GFP cells and analyzed after two days by flow cytometry. Figure 2A gives a typical FACS analysis of NIH3T3-CD4/X4 cells transduced with serially diluted vector supernatants. Titers are given in Figure 2B in infectious units per ml and represent the average values of five experiments. The titers produced by FLY-HIV-87-GFP cells were reproducibly higher than those obtained from FLY-syn-GFP cells. However, the X4R5 phenotype of the 89.6 P HIV-1 Env was retained by the pseudotypes. Only vector particles derived from FLY-syn-GFP cells were able to transduce NIH3T3 cells expressing CD4 and CCR5 (Figure 3).

**Figure 4:**
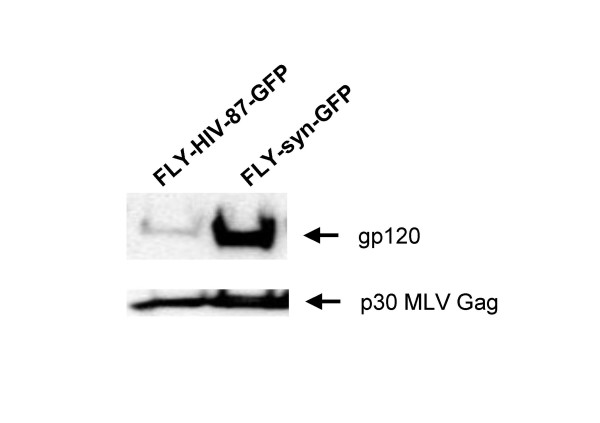
Incorporation of HIV-1 Env into vector particles. Incorporation of Env into MLV/HIV-1 particles was analyzed by Western blot analysis of particles concentrated from FLY cell supernatants by ultracentrifugation. Equal loading was confirmed after stripping the blot and incubating with an anti-MLV-Gag (p30) antibody.

The changed codon usage of the 89.6 P Env resulted in high expression in FLY cells; however, vector titers were always lower than those of vectors containing BH10 Env. To analyze the amount of Env incorporated into particles, the supernatants of the producer cells were collected and centrifuged through a 30% sucrose cushion. The Western blot analysis of the pellets revealed that high levels of the 89.6 P Env was incorporated into vector particles (Figure 4). Equal loading was verified by detection of the MLV p30 Gag protein. These data imply that the amount of Env in viral vector particles does not correlate with their titer. Instead, it seems likely that the fusion ability of Env, which displays strain-specific differences, affects vector infectivity.

### Evaluation of HIV-1 entry inhibitors

Attachment and entry of HIV-1 into CD4 cells involve a series of conformational changes in Env which allow co-receptor binding and finally fusion of viral and cell membranes. AMD-3100 is a small molecule inhibitor of gp120 attachment to the CXCR4 receptor, and T-20 is a synthetic peptide corresponding to a helical region of HIV-1 gp41 that blocks fusion of the cellular and the viral membrane. While AMD-3100 is only active against X4 and X4R5 HIV-1 strains, T20 inhibits fusion of most HIV-1 strains.

**Figure 5:**
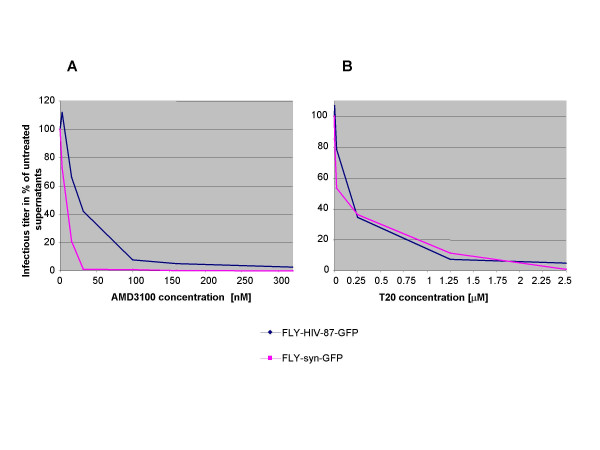
Inhibition of MLV/HIV-1 mediated gene transfer by HIV-1 entry inhibitors. Indicated amounts of either (A) AMD-3100 or (B) T20 were added to vector supernatants during the transduction of NIH3T3 cells expressing CD4 and CXCR4. Transfer of the *gfp *gene was measured and titers are given as percentage of those obtained from untreated supernatants. Inhibition of MLV/HIV-1 vectors was done 2–3 times and had the same outcome. Inhibition of transduction of vectors derived from FLY-HIV-87-GFP cells by AMD3100 was done only once.

As shown in Figure 5A, the sensitivity of the BH10 and 89.6 P Env-containing vectors to AMD-3100 was slightly different. BH10 was less sensitive, indicating a higher affinity for CXCR4. However, the responsiveness to T20 was not significantly different between both Env proteins (Figure 5B). The inhibitor concentrations used in these studies were higher than those previously published because C-terminally truncated Envs have a fast fusion kinetic and are thus less sensitive to entry inhibitors [7]. It has been shown that the cytoplasmic tail slows the folding of HIV-1 Env from a late prebundle configuration into the six-helix bundle, and thereby also slows down the fusion process [8]. Inhibition of HIV-1 Env mediated transduction was specific, since amphotropic MLV could not be inhibited by AMD3100 or T20 (data not shown).

We present here two producer cell lines that release MLV/HIV-1 pseudotyped retroviral vectors particles. Transfer of the *gfp *gene can be used as an indication of an infection process mediated by the HIV-1 envelope glycoprotein. This assay is robust and simple to perform and GFP expression can be rapidly monitored by flow cytometry without further staining of the target cells. Expression of the HIV-1 Env from a bicistronic vector allowed fast establishment of stable producer cell lines. Optimization of HIV-1 Env codon usage led to high expression without the need for Rev co-expression and will, in combination with the bicistronic vector, facilitate the easy exchange of Env sequences. The system can also be applied to transient vector production, and synthetic genes will permit fast testing of diverse HIV-1 Envs, including those from drug resistant strains.

## Conclusion

MLV/HIV-1 vectors are a valuable screening system for entry inhibitors or neutralizing antisera generated by vaccines.

## Methods

### Plasmids

The truncated variant of the envelope glycoprotein HIV-1 Env Tr712 [1] was derived from the plasmid pLßAc/env-Tr712-neo as a 3.1 kb *Sal*I/*Xho*I fragment and was cloned into the *Xho*I site of the bicistronic vector pEF-IRES-P [9]. The sequence of the 89.6 P HIV-1 Env isolate (aa 1 – 712) was chemically synthesized and the codons were modified to high GC content without changing the coding sequence. The Env signal peptide sequence was exchanged with that of the CD5 receptor. Env was excised as an *Eco*RI/*Xho*I fragment, blunt-ended and cloned into the *Xho*I site of the vector pEF-IRES-P, resulting in the clone pEF-IRES-P-89.6 P.

### Cells

NIH 3T3 derivatives [10], 293T (ATCC #CRL-11268) and FLY [11] cells were grown in Dulbecco's modified Eagle's medium (GIBCO BRL, Eggenstein) supplemented with 10% fetal bovine serum, 1% penicillin/streptomycin and 1% L-glutamine. FLY cells are based on human HT1080 cells and express the MLV Gag/Pol gene product [11]. Stably transfected FLY-HIV-87-GFP or FLY-syn-GFP cells were grown in the medium described above supplemented with 2.5 μg/ml puromycin (Sigma, Deisenhofen).

### Stable transfection of cells

FLY cells (10^6^) were seeded in a 10 cm tissue culture plate. The following day, cells were transfected with 10 μg of the bicistronic construct pEF-IRES-P-89.6 P. Transfection was performed with SuperFect™ Transfection Reagent (Qiagen, Hilden). Forty-eight hours after transfection, 2.5 μg/ml puromycin was added to the medium. After the selection and isolation of single cell clones, cells were tested for HIV-1 envelope glycoprotein expression by Western blot analysis and the best expressing clone was transduced with VSV-G pseudotyped retroviral vectors encoding GFP (pMX-EGFP) [12].

### Retroviral transduction and titer determination

Serial dilutions of vector supernatants from packaging cells were passed through 0.45-μm filters and incubated with 1 × 10^5 ^NIH 3T3-CD4/CXCR4 or NIH 3T3-CD4/CCR5 cells for 6 h and longer incubation (e.g. over night) did not change the titer. Supernatants were mixed with different amounts of T20 (generous gift of Prof. von Laer, Georg-Speyer-Haus, Frankfurt) or AMD-3100 (NIH AIDS Research and Reference Reagent Program) for inhibition assays. The numbers of GFP-expressing cells were detected by FACS analysis 48 – 72 hours after transduction. The titers are given in infectious units per ml (IU/ml) and were determined by calculating the percentage of GFP-positive cells. GFP expression was monitored by a shift to green fluorescence (FL-1). FACS analysis was performed with a FACScan (Becton Dickinson, Heidelberg) using the Cellquest software.

### Preparation of viral proteins and immunoblots

For the analysis of incorporation of Env proteins into the vector particles, supernatant of producer cells was filtered through a 0.45-μm filter (Greiner, Frickenhausen, Germany) and centrifuged through a 30% sucrose cushion for 90 min at 26,000 rpm in an SW28 rotor. The pellet was resuspended in 50 μl SDS loading buffer [13] and 25 μl were analyzed. Cell lysates were prepared as described before [1] and 40 μg protein were analyzed. Proteins were separated by electrophoresis through a 7.5% polyacrylamide gel and electroblotted onto a nitrocellulose transfer membrane (Schleicher & Schuell, Dassel, Germany). Immunostaining was performed in Tris base saline (pH 7.4) with 5% milk powder and 0.1% Tween 20. Proteins were detected by incubation with goat antiserum against gp120 (Dunn Labtech, Ansbach, Germany) or a polyclonal antiserum directed against MLV p30 [1], followed by an incubation with a horseradish peroxidase-labeled anti-goat antibody or protein A. Protein bands were finally visualized by enhanced chemiluminescence detection using the ECL kit (Amersham, Braunschweig, Germany) according to the manufacturer's instructions.

## Competing interests

The author(s) declare that they have no competing interests.

## Authors' contributions

Sandra Siegert and Sonja Thaler performed the experiments, Ralf Wagner and Barbara Schnierle participated in the design of experiments, and Barbara Schnierle oversaw the conduction of the experiments and the interpretation of the results.
